# Seasonal variations in fatigue in persons with rheumatoid arthritis: a longitudinal study

**DOI:** 10.1186/s12891-016-0911-4

**Published:** 2016-02-04

**Authors:** Caroline Feldthusen, Anna Grimby-Ekman, Helena Forsblad-d’Elia, Lennart Jacobsson, Kaisa Mannerkorpi

**Affiliations:** Department of Rheumatology and Inflammation Research, Institute of Medicine, Sahlgrenska Academy, University of Gothenburg, Gothenburg, Sweden; The University of Gothenburg Centre for Person-Centred Care (GPCC), Gothenburg, Sweden; Department of Public Health and Community Medicine, Sahlgrenska Academy and University Hospital, University of Gothenburg, Gothenburg, Sweden; Department of Public Health and Clinical Medicine, Rheumatology, Umeå University, Umeå, Sweden; Section of Physiotherapy, Institute of Neuroscience and Physiology, Sahlgrenska Academy, University of Gothenburg, Gothenburg, Sweden

**Keywords:** Fatigue, Rheumatoid arthritis, Longitudinal study, Seasons, Outcome measures

## Abstract

**Background:**

Fatigue is a prominent symptom in persons with rheumatoid arthritis (RA). Although this symptom has been described to vary in duration and frequency little is known about fluctuations in fatigue over time and season. The aim of this study was to describe monthly and seasonal variations in fatigue, in persons with RA of working age.

**Methods:**

Sixty-five participants diagnosed with RA and aged 20–65 years were recruited from a rheumatology clinic in Sweden. The participants provided self-assessments of their fatigue at seven time points during the four seasons using a 0–100 mm visual analogue scale (VAS) and the Bristol Rheumatoid Arthritis Fatigue Multidimensional Questionnaire (BRAF-MDQ). Multiple regression analysis using mixed models was used to analyze changes in fatigue over time.

**Results:**

The mean ± SD of fatigue rated on the VAS was 51 ± 13, indicating substantial fatigue.

Analysis of monthly variation showed statistically significant variation in fatigue ratings concerning VAS fatigue score (*p* < 0.01) as well as the BRAF-MDQ total score and Living, Cognition (*p* < 0.001), and Physical (*p* < 0.05) sub-scores, but not the BRAF-MDQ Emotional sub-score. The greatest variations were seen from January to September, with higher fatigue ratings in January. The changes in VAS fatigue scores over time were considered to be of clinical importance. Analysis of seasonal variation revealed a statistically significant seasonal variation in fatigue levels, with higher fatigue values during the winter as measured by VAS fatigue score (*p* < 0.01) as well as BRAF-MDQ total score (*p* < 0.01) and Physical and Living sub-scores (both *p* < 0.01). The greatest variation was seen between winter and autumn for VAS fatigue and between winter and summer for BRAF-MDQ total score and Physical and Living sub-scores. There were no statistical differences in fatigue levels, monthly or seasonal, between sexes or age groups.

**Conclusions:**

The majority of rating scales used in this study showed fluctuations in fatigue, general and physical fatigue being significantly greater during the winter. As fatigue is a substantial symptom in many persons with RA, this information is important for rheumatology professionals when dealing with persons with RA in routine care.

## Background

Fatigue has high priority for persons with rheumatoid arthritis (RA) [1–3], and besides pain is cited as the most prominent symptom of the disease [4–6]. The reported prevalence of fatigue in RA varies from 42 to 80 % [4, 5, 7, 8]. Severe fatigue is reported by about 50 % of persons with RA [7, 8].

Persons with RA describe fatigue as multidimensional, overwhelming, and unpredictable, with physical, cognitive, and emotional components. The fatigue associated with RA is distinct from normal fatigue in that it is often extreme, unexpected, and not restored by sleep; as such, it affects everyday tasks and social roles [3, 6, 9].

Fatigue impacts individuals differently, and management strategies vary [10]. Women report higher levels of fatigue than men [4, 11, 12], in particular younger women who have to undertake multiple daily roles [10]. Persons of working age are likely to have multiple daily roles and high demands in life, and are thereby more vulnerable to the impact of fatigue [10]. The duration and frequency varies among individuals [3], with studies reporting increasing fatigue as the day passes [13, 14]. Inconsistent results regarding changes in fatigue have been reported in longitudinal studies based on a single baseline assessment and a single follow-up assessment 1 year later. Applying this method, some studies found stable fatigue levels [8, 15] and others found variations [16]. When studying possible fluctuations in fatigue, it appears evident that fatigue should be assessed more frequently than twice a year. Previous studies also indicate inconsistent results regarding seasonal variations in RA; a Japanese study reported seasonal variations in disease activity [17], while a Canadian study found no seasonal variation in pain and global severity [18]. To our knowledge, no longitudinal study has investigated seasonal variations in fatigue in persons with RA.

As fatigue negatively impacts life in persons with RA, the Outcome Measures in Rheumatology Clinical Trials (OMERACT) group recommends that measurements of fatigue should be included in RA clinical trials [2]. Traditionally, fatigue is measured by single items assessing general fatigue, such as a one-dimensional visual analogue scale (VAS). Other instruments measure multiple aspects of fatigue including consequences and impacts. Such multi-dimensional fatigue measures incorporate sub-scores measuring, for example, physical and cognitive aspects of fatigue [19]. However, little is known about fluctuations in fatigue over time and season as measured by general VAS ratings and multi-dimensional rating scales. A recent review article concluded that most studies of fatigue are cross-sectional, and there is a need for longitudinal studies that measure fatigue adequately and regularly over time [20].

The aim of the present study was to investigate variations in fatigue levels reported by persons with RA of working age at seven different time points during the four seasons. Both a single-item and a multi-dimensional fatigue instrument were used to explore: i) how fatigue levels vary over time ii) how the different aspects of fatigue vary and iii) whether there are any seasonal variations in fatigue levels. The analyses were adjusted for sex and age. Our hypothesis is that fatigue fluctuates over time, when measured regularly over one year.

## Methods

### Participants

Study participants were recruited from the hospital administrative register at a rheumatology clinic in Western Sweden. The inclusion criteria were as follows: diagnosed with RA according to the International Classification of Diseases (ICD; diagnosis codes M05 and M06) [21]; of working age (20–65 years); disease duration >3 years; and stable pharmacological treatment with disease-modifying anti-rheumatic drugs (DMARDs), including conventional DMARDs, biological DMARDs, and glucocorticosteroids, for >3 months prior to entry into the study. As the impact of fatigue is suggested to be strongest in persons with multiple social roles [10], persons of working age were selected for this study as they were considered likely to have multiple social roles. Disease duration >3 years was chosen to ensure that the participants had had the possibility to adapt to their RA disease and reach stability in their medication. Individuals were excluded if they presented with other severe somatic or psychiatric diseases, or if they were unable to communicate effectively in Swedish.

### Study design and procedure

**Fig. 1:**
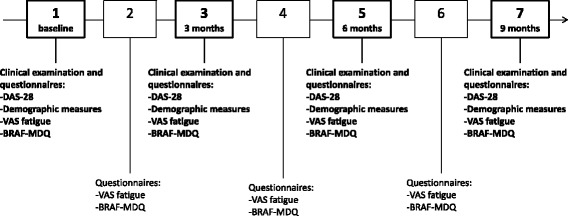
Flowchart of the longitudinal study of variations in fatigue in persons with rheumatoid arthritis

All participants were invited to attend four separate clinical examinations, three months apart, during the course of the study, in order to capture the four seasons. At these examinations, demographic and disease-related data were collected and questionnaires aimed at assessing the level of fatigue were administered. To obtain a nuanced picture of the variations in fatigue levels and to ensure that information was recorded during all four seasons, fatigue assessment questionnaires were also sent to the participants between clinical examinations. The participants thus filled in fatigue assessment questionnaires at seven time points during the course of the study (Fig. 1).

### Measures of fatigue

Baseline demographic and disease-related measures were age, sex, work status, and disease duration. Disease activity was assessed using Disease Activity Score (DAS-28) [22], which includes tenderness and swelling in 28 joints, patient-rated global health assessed by a 0–100 mm VAS, and erythrocyte sedimentation rate. The participants also self-reported pain levels using a VAS (0–100 mm with anchors of “no pain” and “worst imaginable pain”) and physical function using the Swedish version of the Health Assessment Questionnaire (HAQ). The HAQ score ranges from 0 to 3 [23, 24].

The VAS, a single-item measure, was used to assess general fatigue (0–100 mm) during the last week (anchors: “no fatigue” and “worst imaginable fatigue”) [25, 26]. Scores >50 were considered to indicate severe fatigue [7]. The Swedish version of the Bristol Rheumatoid Arthritis Fatigue Multidimensional Questionnaire (BRAF-MDQ) [27, 28], was used to measure multi-dimensional RA-specific fatigue during the last 7 days. The BRAF-MDQ comprises a total (summary) score (0–70) and four sub-scores: Physical (0–22; a measure of physical fatigue), Living (0–21; describing sequelae due to the unpredictability of fatigue), Cognition (0–15; describing the cognitive effects of fatigue, such as errors and/or a lack of concentration), and Emotion (0–12; describing the effects of fatigue on emotions and mood). A higher score denotes more severe fatigue.

### Statistical analysis

Descriptive data for demographics were calculated as mean ± standard deviation (SD) and median (max;min) for continuous variables and as numbers (*n*) and percentages for categorical variables.

#### Average fatigue over one year

For each participant, the mean ± SD was calculated for each of the different fatigue measures across each of the seven assessments. These individual mean ± SD values were than expressed as a group mean ± SD for the whole study population. The *t*-test was used to analyze differences between the sexes, and ANOVA was used to analyze differences between age groups. These statistical analyses were performed using version 15.0 of the SPSS software package (SPSS Inc., IBM, Chicago USA).

*Fatigue levels and monthly and seasonal variations* were analyzed by multiple regression analysis with mixed models. PROC MIXED within version 9.3 of the SAS software package (SAS Institute Inc.) was applied, with “month” (numbers of observations: December *n* = 26; January *n* = 57; February *n* = 37; March *n* = 32; April *n* = 40; May *n* = 46; June *n* = 25; July *n* = 48; August *n* = 33; September *n* = 32; October *n* = 9; November *n* = 41) and “season” (winter *n* = 120; spring *n* = 118; summer *n* = 106; autumn *n* = 82) as outcome variables. The seasons were defined as follows: winter: December, January, and February; spring: March, April, and May; summer: June, July, and August; and autumn: September, October, and November. As both sex and age are suggested to be associated with fatigue, sex (female/male) and age groups (<47, 47–55, 56–60, and >60, with an equal number of participants assigned to each group) were included in the regression model. A random intercept model was used for analysis. The fixed-effects variables included in the first version of the model were month, sex, and age group, and those included in the second version of the model were season, sex, and age group. The results from the analysis of fatigue over time (measured in months) are presented as a separate figure for each outcome. These figures are based on the model means, the so called LSMEANS, and their 95 % confidence intervals. Participants who completed more than 50 % (≥4 of 7) of the assessments were included in the analyses.

The study was approved by the Regional Ethical Review Board in Gothenburg. Participants received both written and oral information, and all provided written consent.

## Results

### Participants

Persons with RA who had visited the rheumatology clinic at Sahlgrenska University Hospital during the last 1.5 years, who had a diagnosis code, and who fulfilled the age criterion, were identified from the clinic’s administrative register (*n* = 1627). Of these, 250 persons were randomly selected using a computerized randomization list and checked against the inclusion and exclusion criteria by reviewing the medical records. Eligible persons were sent a letter and an invitation to participate in the study (*n* = 140). Of these, 72 persons responded and 65 persons entered into the study; seven declined to participate, citing health problems (*n* = 3), a lack of time (*n* = 2), family reasons (*n* = 1), or the amount of effort required by the study (*n* = 1). Three of the participants were aged 65 years when recruited, but had turned 66 by the time they entered the study. Overall, 91 % (*n* = 61) of the participants participated in >50 % of the assessments: 7 assessments (*n* = 51); 6 assessments (*n* = 6); 5 assessments (*n* = 2); 4 assessments (*n* = 2); 3 assessments (*n* = 1); 2 assessments (*n* = 3). The reasons for missing assessments were time limitations (*n* = 6), illness (*n* = 4), and incomplete questionnaires (*n* = 4). Baseline characteristics of the 65 persons with RA enrolled in the study are presented in Table 1.

**Table 1:** Demographic data for the 65 persons with rheumatoid arthritis

Variable		mean ± SD median (max;min)	*n* (%)
Age (years)		54 ± 9.9	
		56 (23;66)	
Sex, female			48 (74 %)
Disease duration (years)		15 ± 9.6	
		12 (4;45)	
DAS-28 (score)		3.7 ± 1.4	
		3.8 (0.8;6.9)	
Tender joints (n)		6.7 ± 6.3	
		5 (0;27)	
Swollen joints (n)		3.9 ± 4.1	
		3 (0;21)	
Erythrocyte sedimentation rate (mm)		10.9 ± 10.2	
		7 (2;53)	
Global, VAS 0–100 (mm)		36 ± 22.7	
		32 (1;83)	
Pain, VAS 0–100 (mm)		38 ± 25.5	
		34 (0;100)	
HAQ (score)		0.6 ± 0.6	
		0.6 (0;2.4)	
Medication			
	No DMARD		8 (12 %)
	Conventional synthetic DMARD		57 (88 %)
	Biological DMARD		15 (23 %)
	Corticosteroids		7 (11 %)
Work status			
	Working or studying (full-time or part-time)		41 (63 %)
	Unemployed		7 (11 %)
	Retired		6 (9 %)
	Disability benefits (full-time or part-time)		24 (37 %)
	Parent’s allowance		2 (3 %)

*n* number, *VAS* visual analogue scale, *DAS* Disease Activity Score, *HAQ* Health Assessment Questionnaire, *DMARD* disease-modifying anti-rheumatic drug

### Baseline fatigue levels

Baseline fatigue levels displayed a large variation, ranging from 0 to 92 on the VAS (0–100) and from 0 to 65 on the BRAF-MDQ total score (0–70). The BRAF-MDQ also showed a large range of ratings for all sub-scores: 0–21 for Physical (0–22), 0–21 for Living (0–21), 0–15 for Cognition (0–15), and 0–12 for Emotion (0–12).

### Average fatigue over the course of the study

**Fig. 2:**
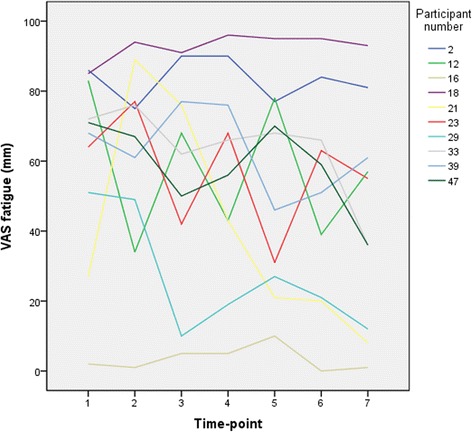
Fluctuation in general fatigue (VAS fatigue) over the course of the study in 10 participants included in the study during the same season and selected to this graph to illustrate the variability of fatigue fluctuation among individuals. VAS = visual analogue scale

The individual measures of fatigue at the seven assessments of the 61 participants participating in >50 % of the assessments in this study showed a wide range: 1–81 mm for the VAS, 2–48 for the BRAF-MDQ total score, 1–16 for BRAF-MDQ Physical, 0–15 for BRAF-MDQ Living, 0–12 for BRAF-MDQ Cognition, and 0–10 for BRAF-MDQ Emotion. Figure 2 illustrates the fluctuation in fatigue over time, with VAS fatigue ratings in 10 participants selected to illustrate the diversity in fatigue fluctuation. The mean general fatigue rated by the single-item VAS at the seven assessments during the study period was 51 ± 13 mm (see Table 2). To standardize the BRAF-MDQ sub-score ratings, percentages of the maximum score of each sub-score were calculated. The ratings on the Physical sub-score indicated severe fatigue (61 % of maximum score) while the ratings in the other dimensions yielded lower levels; 28 % of maximum for Living, 33 % for Cognition, and 28 % for Emotion (see Table 2). The results (mean ± SD) are presented in Table 2 both for the total population and after stratification according to sex and age group.

**Table 2:** Average fatigue over the course of the study rated on VAS and BRAF-MDQ, presented as mean ± SD of the individual fatigue scorings at the seven time points for the total population and after stratification according to sex and age group

Measure		Total (*n* = 61)	Women (*n* = 47)	Men (*n* = 14)	Age 20–46 (*n* = 15)	Age 47–56 (*n* = 15)	Age 57–61 (*n* = 15)	Age 62–66 (*n* = 16)
VAS fatigue (0–100)	mean ± SD	51.2 ± 12.7	54.6 ± 13.1	39.6 ± 11.5	45.7 ± 10.7	56.3 ± 14.7	56.9 ± 13.3	46.0 ± 12.2
BRAF-MDQ								
Total (0–70)	mean ± SD	27.0 ± 6.4	28.6 ± 6.4	21.6 ± 6.3	22.7 ± 6.4	29.5 ± 7.0	31.5 ± 6.1	24.3 ± 5.9
Physical (0–22)	mean ± SD	13.5 ± 2.4	14.0 ± 2.4	11.8 ± 2.3	12.5 ± 2.3	15.0 ± 2.8	14.0 ± 2.2	12.5 ± 2.3
Living (0–21)	mean ± SD	5.8 ± 2.2	6.2 ± 2.2	4.7 ± 2.0	5.4 ± 2.3	5.4 ± 2.2	7.8 ± 2.3	4.9 ± 1.8
Cognition (0–15)	mean ± SD	5.0 ± 1.9	5.1 ± 1.9	4.6 ± 1.9	4.2 ± 1.9	4.8 ± 2.0	6.4 ± 1.8	4.5 ± 1.8
Emotion (0–12)	mean ± SD	3.4 ± 1.5	3.5 ± 1.5	2.8 ± 1.4	2.5 ± 1.3	4.2 ± 1.7	4.2 ± 1.2	2.5 ± 1.6

*VAS* visual analogue scale, *BRAF-MDQ* Bristol Rheumatoid Arthritis Fatigue-Multidimensional Questionnaire, *SD* standard deviation, *n* number

The mean fatigue scores after stratification according to sex and age are presented in Table 2. Women reported higher numerical fatigue values for all fatigue measures. The differences in BRAF-MDQ scores between women and men were not statistically significant but the VAS scores differed significantly between sexes (*p* < 0.05). In relation to age, younger (20–46 years) and older (62–66 years) participants recorded lower numerical fatigue scores than the other two groups (47–56 and 57–61 years of age), although the differences were not statistically significant for either the VAS scores or the BRAF-MDQ scores. The proportions of women and men in each age group were similar.

### Fatigue levels and monthly variations

**Fig. 3:**
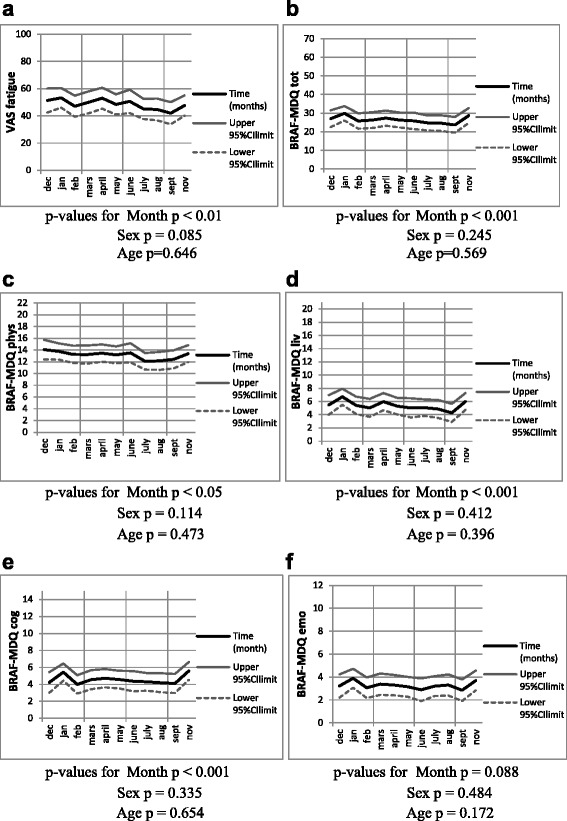
Monthly variations in fatigue rated by the VAS and BRAF-MDQ total and sub-scales of fatigue. Statistical analysis was performed with mixed models including sex and age. Number of observations (n): Dec *n* = 26, Jan *n* = 57, Feb *n* = 37, Mar *n* = 32, Apr *n* = 40, May *n* = 46, Jun *n* = 25, Jul *n* = 48, Aug *n* = 33, Sep *n* = 32, Oct *n* = 9*, Nov *n* = 41. *Due to the low number of observations in October, the estimate for this month was omitted from the graphs

Statistically significant monthly variations were seen in five of the six fatigue measures: the VAS fatigue scores (*p* < 0.01), the BRAF-MDQ total scores, and the Living, Cognition (*p* < 0.001), and Physical (*p* < 0.05) sub-scores. The greatest variation was seen from January to September for all five of these, except for the Physical sub-score where the greatest variation was between December and July. The highest fatigue values were recorded in January or December. Change in the Emotion sub-score did not reach a statistically significant level (*p* = 0.09). Monthly variations did not differ significantly according to sex or age group (Fig. 3).

### Fatigue levels and seasonal variations

Analysis of seasonal variations in the fatigue ratings showed a statistically significant seasonal variation for the single-item general VAS fatigue score (*p* < 0.01) and the BRAF-MDQ total score (*p* < 0.001), as well as the two dimensions covering physical aspects (Physical and Living; *p* < 0.01). The greatest variation was seen between winter and autumn for VAS fatigue and winter and summer for BRAF-MDQ, with the highest values during the winter. No seasonal variation in fatigue levels was observed for the two dimensions covering mental aspects (Cognition and Emotion). Neither age nor sex had significant impact on seasonal variations in fatigue levels (Table 3).

**Table 3:** Seasonal variations in fatigue levels, in persons with rheumatoid arthritis, rated by the VAS for general fatigue and the BRAF-MDQ

Measure			Least square of means	95 % CI	*p*-value
VAS fatigue	Season	Winter	50.86	44.15; 57.57	<0.01
(0–100)		Spring	50.62	43.88; 57.36	
		Summer	46.34	39.53; 53.16	
		Autumn	44.88	37.95; 51.81	
	Sex				0.08
	Age				0.62
BRAF-MDQ					
Total	Season	Winter	28.15	24.52; 31.78	<0.01
(0–70)		Spring	26.77	23.12; 30.43	
		Summer	25.13	21.44; 28.81	
		Autumn	25.59	21.85; 29.33	
	Sex				0.25
	Age				0.56
Physical	Season	Winter	13.67	12.41; 14.94	<0.01
(0–22)		Spring	13.30	12.03; 14.57	
		Summer	12.46	11.18; 13.75	
		Autumn	12.74	11.43; 14.05	
	Sex				0.11
	Age				0.46
Living	Season	Winter	6.11	4.99; 7.24	<0.01
(0–21)		Spring	5.52	4.39; 6.66	
		Summer	5.06	3.92; 6.21	
		Autumn	5.10	3.93; 6.27	
	Sex				0.44
	Age				0.43
Cognition	Season	Winter	4.78	3.85; 5.71	0.40
(0–15)		Spring	4.67	3.73; 5.61	
		Summer	4.32	3.37; 5.27	
		Autumn	4.65	3.69; 5.62	
	Sex				0.34
	Age				0.70
Emotion	Season	Winter	3.54	2.78; 4.31	0.33
(0–12)		Spring	3.29	2.52; 4.06	
		Summer	3.19	2.41; 3.97	
		Autumn	3.15	2.36; 3.94	
	Sex				0.50
	Age				0.16

Statistical analyses was performed using mixed models, including age and sex. Number of observations (n): winter *n* = 120, spring *n* = 118, summer *n* = 106, autumn *n* = 82. VAS = visual analogue scale, BRAF-MDQ = Bristol Rheumatoid Arthritis Fatigue Multidimensional Questionnaire, CI = confidence interval

Patient-rated global health measured by VAS, self-reported pain level measured by VAS and physical function measured by HAQ were assumed to be related to fatigue, and were rated at the four clinical examinations. No seasonal variations were found in any of these measures during the study period (global health: *p* = 0.39; self-reported pain level: *p* = 0.074; physical function: *p* = 0.49).

## Discussion

The results of this longitudinal study revealed statistically significant monthly variations in levels of general fatigue, both rated on the single-item VAS fatigue and rated on the multi-dimensional BRAF-MDQ total score. In addition, participants reported significant monthly variations in the Physical, Living, and Cognitive dimensions of fatigue included in the BRAF-MDQ. We also found a significant seasonal variation, with the most severe fatigue in winter, in the four measurements assessing general and physical dimensions of fatigue. Interestingly, the mental dimensions of fatigue did not show seasonal differences. These results support the use of multi-dimensional measures of fatigue. From the perspective of patients, as well as that of professionals, this information can be helpful in developing strategies to handle fatigue, such as providing adequate information about expected fluctuations in fatigue and suggestions about physical activity during the winter months.

The majority of the participants in this study were either working or studying; this means that they were likely to take a summer vacation, which may have contributed to the lower fatigue scores recorded during the summer and early autumn. The study was carried out in Sweden, a country with large variations in temperature and hours of daylight between winter and summer, and so several factors related to living might have influenced fatigue levels, such as outdoor activities and enjoying nature. Physical activity is inversely associated with fatigue [29, 30], and the level of physical activity has been shown to be highest in spring and summer [31]. This may be one reason why the physical but not the mental aspects of fatigue decreased during summer. Levels of vitamin D might also have had an influence: a British study found that 13 % of people with RA had a vitamin D deficiency, and a further 50 % had unsatisfactory levels of vitamin D during winter [32]. Pain and depressive mood have been suggested to be associated with fatigue [20], but no seasonal variations in pain or depression have been detected in RA [18, 33]. Further research is needed regarding seasonal influence and possible predictors of the variations in fatigue over time.

The changes in fatigue were statistically significant but the clinical value of these changes needs to be considered. The minimal clinically important difference in the 0–100 VAS fatigue score is suggested to be 10 mm [34, 35]. The mean seasonal change was within the 10 mm limit, while in some cases the change between two separate months (in particular, January and September) was larger than 10 mm and considered to be of clinical importance. Regarding the BRAF-MDQ total score, the minimum change to indicate a clinically important improvement is suggested to be 7.43 points, while a decrease of 2.58 points indicates a deterioration [28]. The changes in fatigue in our material were within the 7.43 point limit, and therefore do not indicate a clinically important difference.

The mean value of fatigue rated on the VAS (51 ± 13) was in line with previous research on fatigue in RA [7]. However, the mean fatigue in a large international study was below 40 [36]. The large range of baseline fatigue ratings (0–92) indicates the representativity of the study group. The study population comprised three times more women than men, which corresponds to the prevalence of RA in the general population [37]. Monthly and seasonal variations in fatigue were similar in men and women, although the women tended to report higher numerical fatigue values than men for all fatigue measures. However, due to the relatively low number of men enrolled, caution must be taken when interpreting for differences related to sex.

We also found that age had no significant impact on the levels of fatigue reported over time. Because there was no linear relationship between age and fatigue (Table 2), age was entered into the analysis as a categorical variable (four different age groups) rather than a continuous variable. The age group containing the youngest participants was wider than the others (20–46 years of age), due to the low number of younger participants. The conclusions regarding this group are therefore less precise, and should be studied further. As all participants were of working age, no data were collected for persons older than 65–66 years. Previous studies have reported both significant [38] and non-significant [15] correlations between age and fatigue.

This study has several limitations, such as the small sample size. Another is the lack of a healthy control group; similar time-related changes in fatigue levels may also occur in healthy individuals. Most participants ended their participation in August or September, meaning there were few observations in October (*n* = 9), and so we chose to omit the October estimates from Fig. 3.

One strength of this study lies in the use of a longitudinal design. As measurements were taken at seven time points over the course of the study, we were able to detect variations in fatigue. Levels of severe fatigue were reported by approximately half of the study population, implying that chronic fatigue is a substantial problem in persons with RA. The present study is the first to identify natural variations in fatigue levels over time and according to season in persons with RA. Further studies are needed to search for factors that influence fatigue in RA over time.

## Conclusions

Frequent assessments of fatigue levels in persons with RA revealed statistically significant variations in fatigue in five of the six rating scales used in this longitudinal study. We also identified statistically significant seasonal variations in general fatigue and in the physical aspects of fatigue, both suggesting that persons with RA experience greater fatigue during the winter. This information is important both for rheumatology professionals and for patients, as it implies that time and season may affect fatigue in persons with RA. It also highlights the importance of using multi-dimensional instruments to measure fatigue, in order to better understand the influence of fatigue on daily life.

## Abbreviations

BRAF-MDQ: Bristol rheumatoid arthritis fatigue- multidimensional questionnaire
DAS-28: Disease activity score
DMARDs: Disease-modifying anti-rheumatic drugs
HAQ: Health assessment questionnaire
RA: Rheumatoid arthritis
SD: Standard deviation
VAS: Visual analogue scale
